# Supplementary material for: UhAVR1, an HR-Triggering Avirulence Effector of Ustilago hordei, Is Secreted via the ER–Golgi Pathway, Localizes to the Cytosol of Barley Cells during in Planta-Expression, and Contributes to Virulence Early in Infection
Source: J Fungi (Basel). 2020 Sep 18;6(3):178. doi: 10.3390/jof6030178 (PMC7559581; doi:10.3390/jof6030178)
Supplement: Supplementary file 1 [file jof-06-00178-s001.pdf]

Supplementary Figures

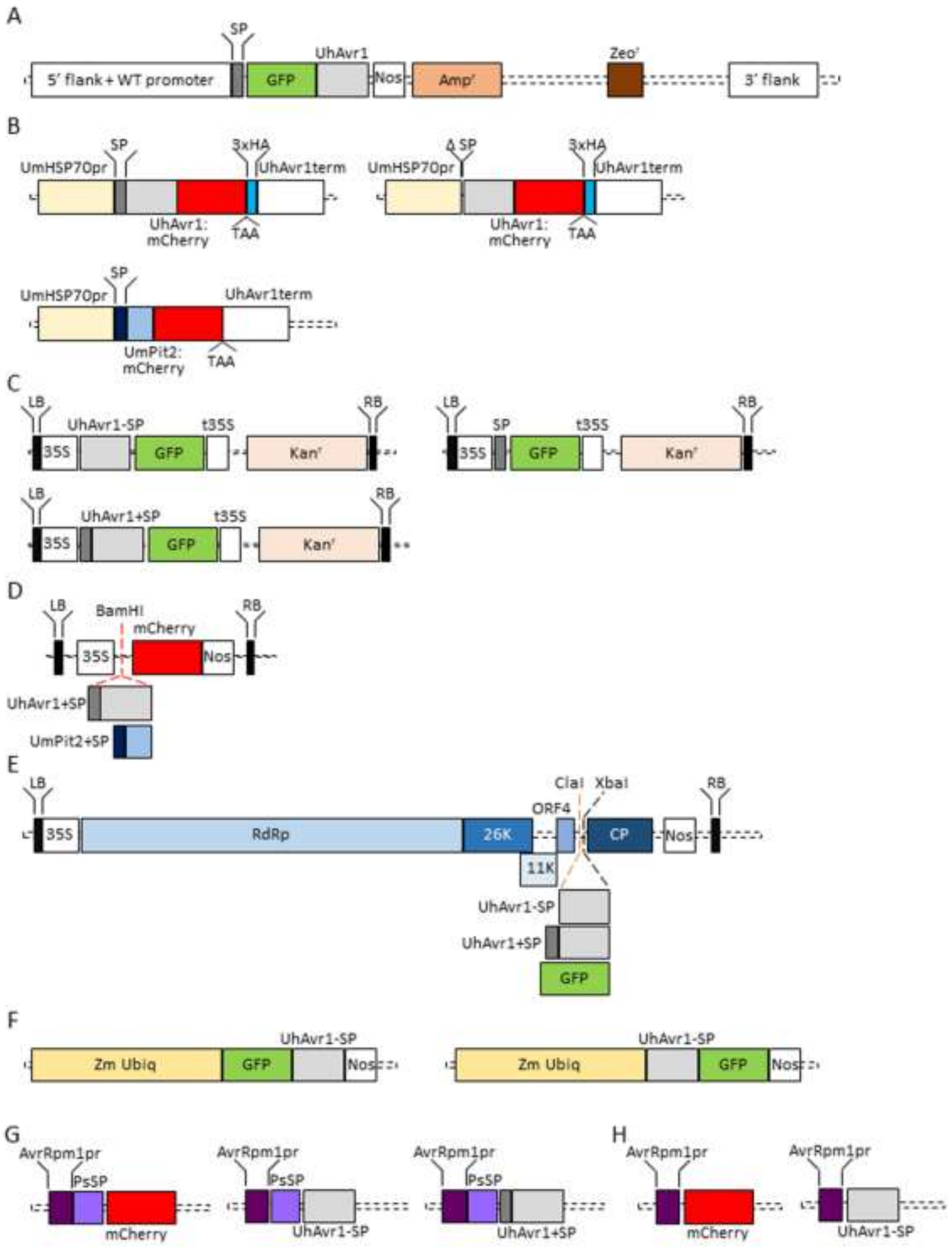

Figure S1. Constructs used during this study. (A) Gene cassette SP:GFP:UhAvr1:Nos used to generate the strain Uh1398 (*MAT-1 ΔUhAvr1* [*SP:GFP:UhAvr1*]). A gene cassette SP:GFP:UhAvr1:Nos was cloned downstream of

sequences for homologous recombination (5′ flank + WT promoter and 3′ flank). **(B)** FunGus expression vectors used in this study expressing UhAvr1+SP:mCherry, ΔSP:UhAvr1-SP:mCherry or UmPit2+SP:mCherry. **(C)** GFP constructs used in barley and *N. benthamiana* transient assays expressing UhAvr1-SP:GFP, UhAvr1+SP:GFP or SP:GFP. **(D)** mCherry constructs used in *N. benthamiana* transient assays expressing mCherry, UhAvr1+SP:mCherry or UmPit2+SP:mCherry. **(E)** FoMV-based VOX system showing the organization of open reading frames (ORFs) for viral proteins and target genes UhAvr1-SP, UhAvr1+SP or GFP. **(F)** Constructs used for cell death assays in *N. benthamiana* expressing GFP:UhAvr1-SP or UhAvr1-SP:GFP. **(G)** Constructs used for *Psa*-barley assays expressing mCherry, UhAvr1-SP or UhAvr1+SP. **(H)** Deleted *Pseudomonas* SP (PsSP) constructs used for *Psa*-barley assays expressing ΔPsSP:mCherry or ΔPsSP:UhAvr1-SP. Abbreviations: Ampicillin resistance gene (Amp^r), Zeocin resistance gene (Zeo^r), nopaline synthase terminator (Nos), *Ustilago maydis* constitutive HSP70 promoter (UmHSP70pr), UhAvr1 terminator (UhAvr1term), 3x HA tag (3xHA), stop codon (TAA), left border (LB), right border (RB) for *Agrobacterium* T-DNA transfer, Kanamycin resistance gene (Kan^r), CaMV 35S promoter (35S), CaMV 35S terminator (t35S), viral polymerase (RdRp), viral movement proteins (26K, 11K and ORF4), viral coat protein (CP), maize ubiquitin promoter (Zm Ubiq) and *Pseudomonas AvrRpm1* promoter (AvrRpm1pr).

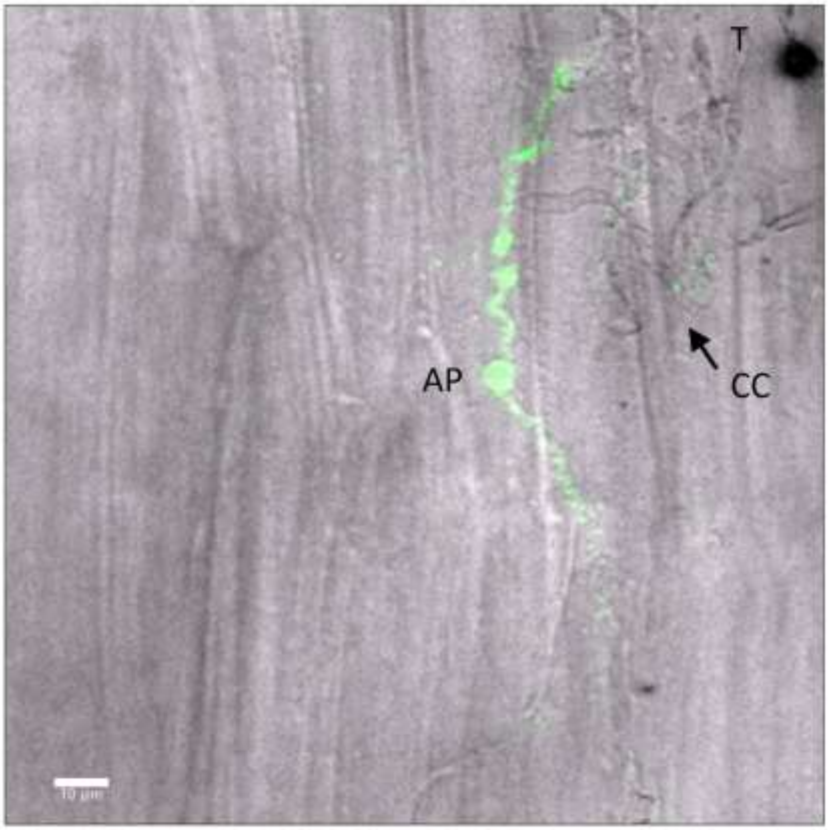

Figure S2. Fungal structure observed during the infection with *Ustilago hordei* teliospores on barley coleoptiles. Barley coleoptiles were infected with UhAvr1m teliospores, stained with WGA-AF488 and PI and visualized with confocal microscopy. An appressorium-like (AP) structure was seen near the junction between two neighbouring barley cells. Fungal hyphae with collapsed cells (CC) and a black teliospore (T) were also visible near the appressorium-like structure. Image is a snapshot of a single z-stack observed at 96 hpi in cv. Hannchen and scale bar represents 10 μm.

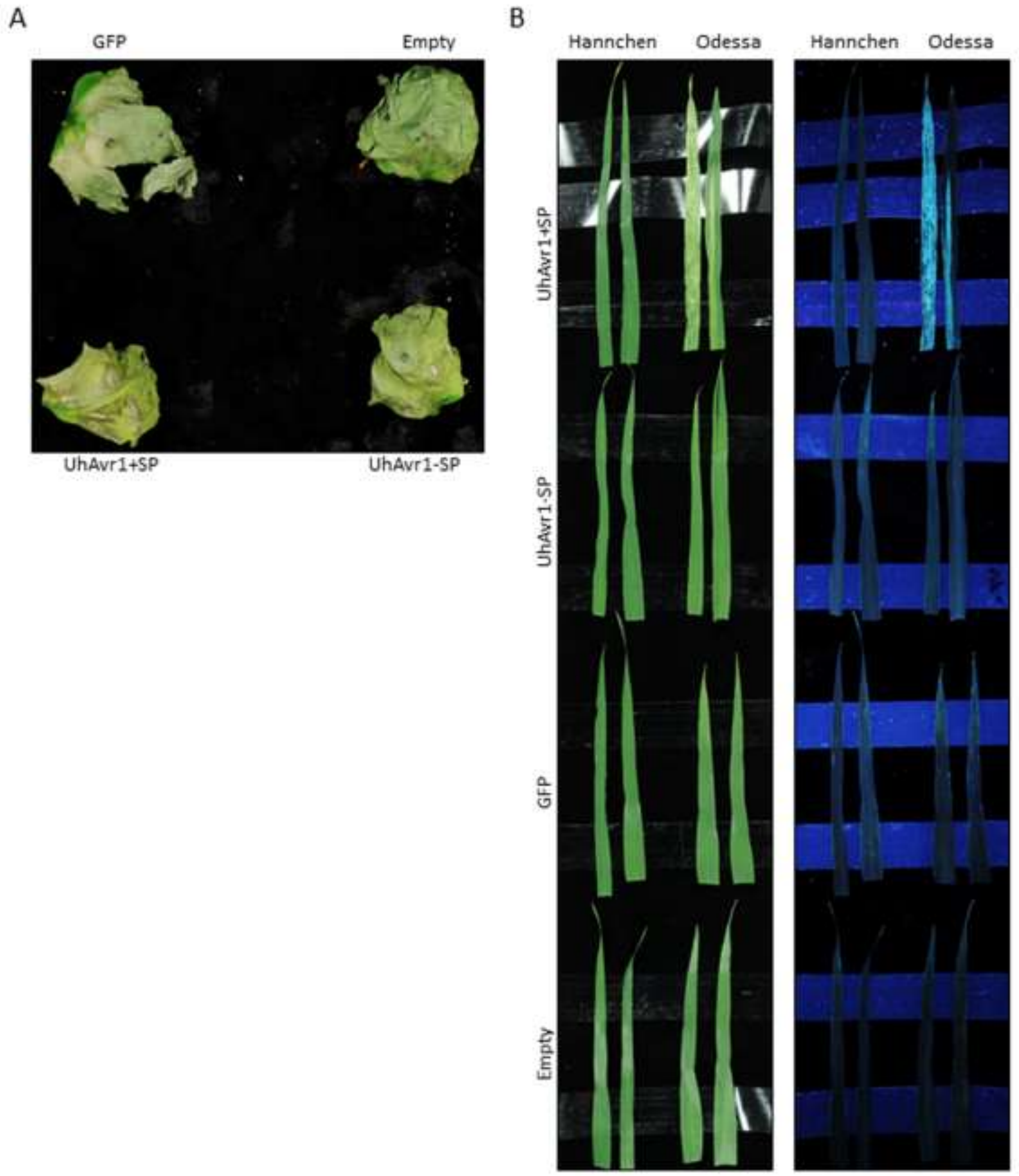

Figure S3. Symptoms on the leaves of *N. benthamiana* and barley plants expressing VOX constructs at late stages. **(A)** Agro-infiltrated leaves of *N. benthamiana* with VOX constructs expressing GFP, UhAvr1-SP, UhAvr1+SP or an empty-vector control showed necrosis at 13 dpi. The experiment was repeated three times with similar results and a representative image is shown. **(B)** Symptom development on the L3 leaves of barley. L1 and L2 leaves of barley cultivars were rub-inoculated with *N. benthamiana* leaf sap previously infected with the VOX constructs as indicated. At 15 dpi, a few plants of cv. Odessa expressing UhAvr1+SP showed mosaic symptoms on their L3 leaves. The images were taken under white light (left) or long-wavelength UV light (right) from the same set of leaves. The experiment was repeated three times with similar results and a representative image is shown.

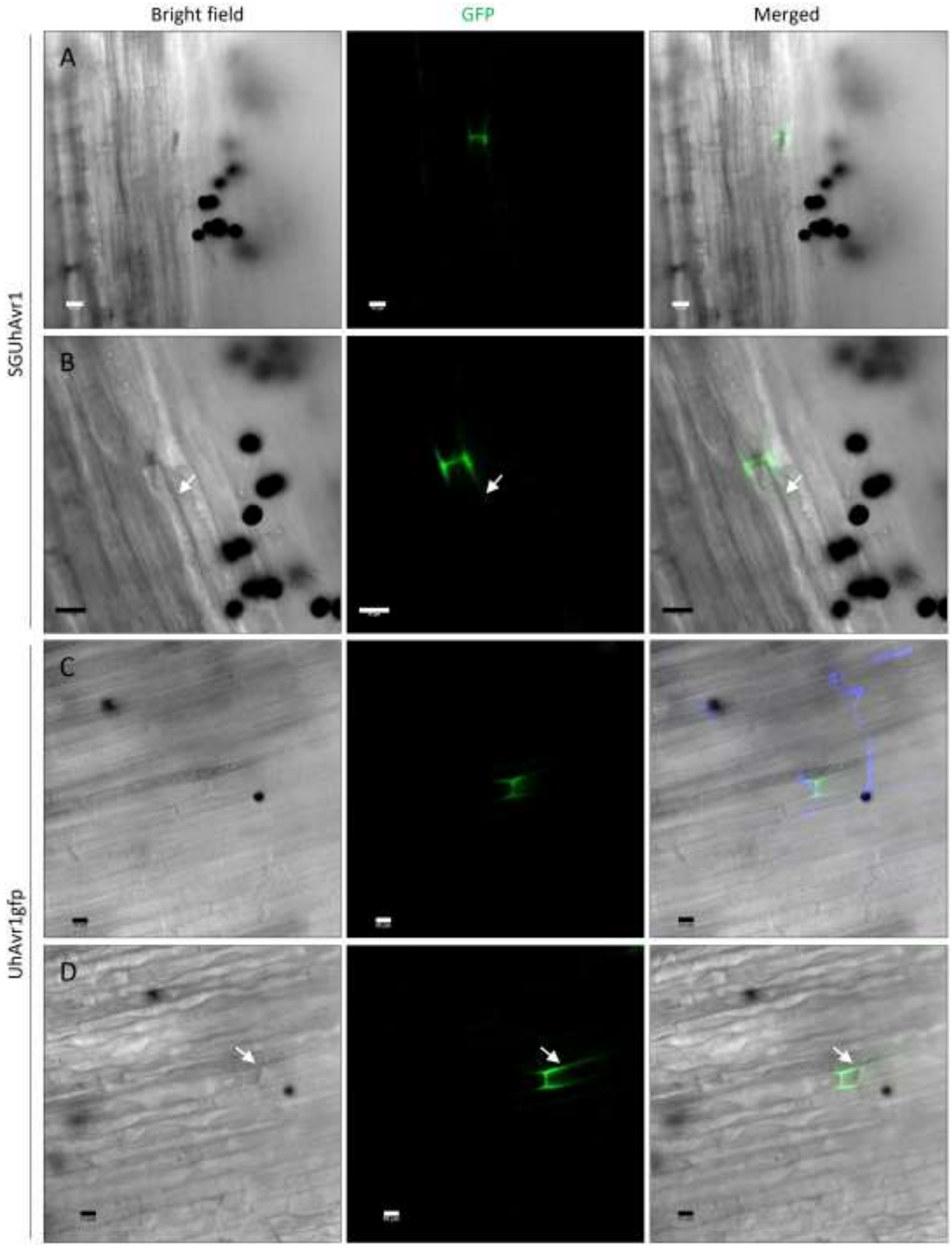

Figure S4. GFP fluorescence resulting from inoculation with *U. hordei* strains expressing GFP-tagged UhAVR1 localizes to the intercellular space of cv. Odessa cells. Confocal imaging of cv. Odessa seedlings infected with SGUhAvr1 (Uh362 (*MAT-2 Uhavr1*) x Uh1398 (*MAT-1 ΔUhAvr1* [*SP:GFP:UhAvr1*])) **(A and B)** or UhAvr1GFP (Uh362 (*MAT-2 Uhavr1*) x Uh1357 (*MAT-1 ΔUhAvr1* [*otef:UhAvr1:GFP*])) **(C and D)** teliospores expressing N-terminally or C-terminally GFP-tagged UhAVR1, respectively, revealed the GFP fluorescence in the intercellular space of barley cells near the infection sites. Fungal hyphae were stained with Uvitex 2B (purple) in panel C. **(B and D)** GFP fluorescence remained associated with the cell border even after performing plasmolysis with 1-1.5M NaCl (arrow points to the retracted plasma membrane). All images are snapshots of a single optical section and the scale bar represents 10 μm.

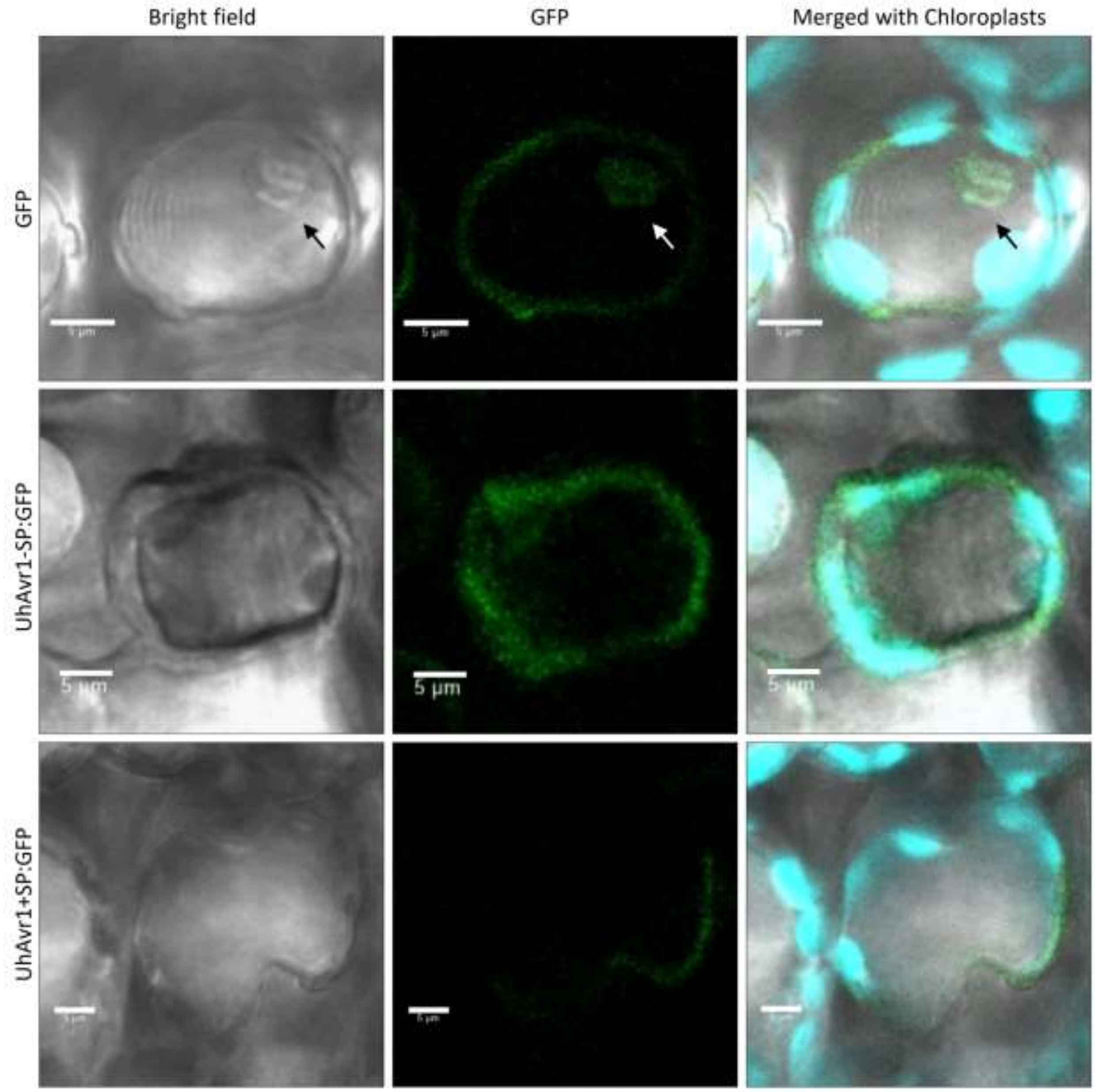

Figure S5. **UhAVR1:GFP localizes to the cytosol of cv. Odessa cells at 48 hpi.** Confocal microscopy of leaves of cv. Odessa agroinfiltrated with UhAvr1-SP:GFP or UhAvr1+SP:GFP displayed GFP fluorescence localizing to the cytosol, surrounding the chloroplasts. Whereas, the free GFP control showed fluorescence localizing to the nucleoplasm (arrow) and the cytosol. The same GFP constructs were used for barley protoplast transfection (Figure 7) and *N. benthamiana* agro-infiltrations (Figure 8). All images are snapshots of a single optical section and the scale bar represents 5 µm.

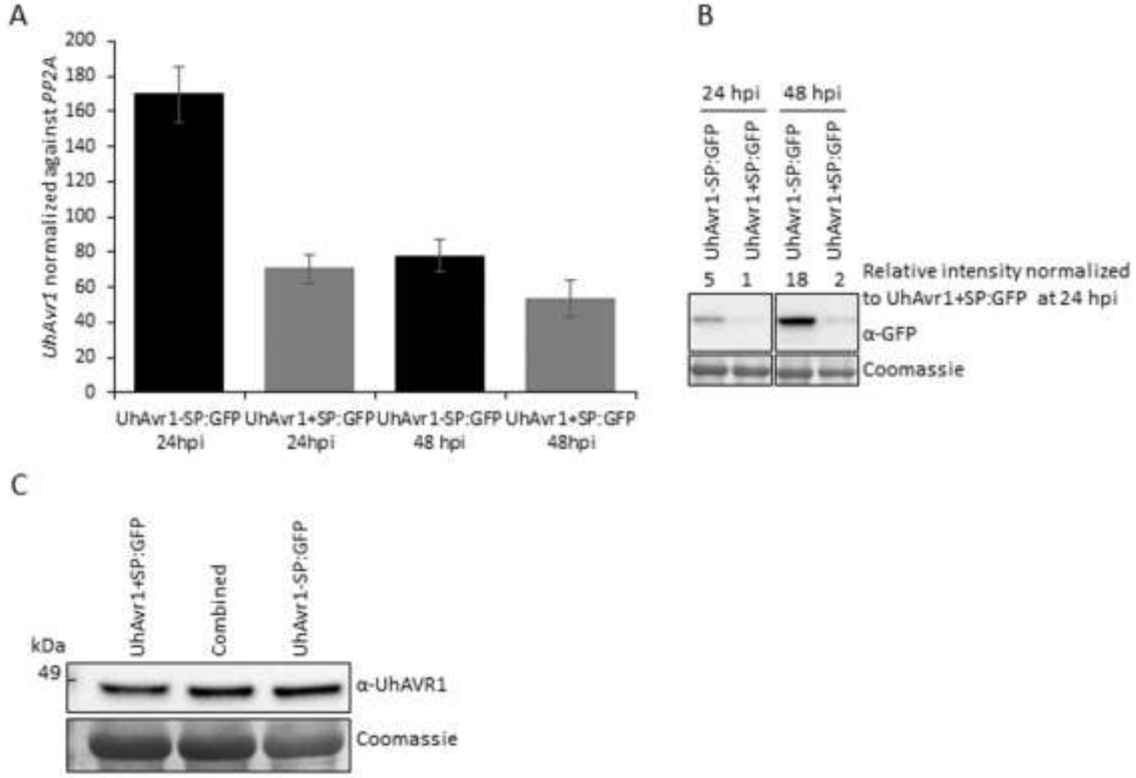

Figure S6. Transcript and protein accumulation of UhAVR1 is affected by the presence of a SP and the SP is cleaved off from the pre-protein in *N. benthamiana*. (A) Quantification of *UhAvr1* transcripts by ddPCR from agroinfiltrated *N. benthamiana* plants expressing UhAvr1-SP:GFP and UhAvr1+SP:GFP at 24 and 48 hpi, with corresponding protein accumulation from one of the experimental repeats (B). Normalization of *UhAvr1* transcripts was done against the *N. benthamiana* gene *PP2A*. The ddPCR graph shows the average of two experimental repeats, each including three technical repeats, along with their standard deviation depicted as error bars. **(B)** Protein blot analysis on agro-infiltrated *N. benthamiana* at 24 and 48 hpi, corresponding to one of the repeats used in panel A. The intensity value of each protein band detected by anti-GFP antibody was calculated by Image Lab software, normalized to UhAVR1+SP:GFP expression at 24 hpi and is provided on the top of the gel picture. Lower levels of protein as well as transcripts from UhAvr1+SP:GFP compared to UhAvr1-SP:GFP were detected at 24 and 48 hpi. **(C)** Products resulting from the agroinfiltration of UhAvr1+SP:GFP and UhAvr1-SP:GFP were loaded individually (left and right lane) or combined (middle lane) to determine the possible size differences between these two proteins. The sample volumes were adjusted in all three lanes by adding protein lysates from a healthy *N. benthamiana* plant to avoid discrepancies in migration of proteins due to unequal protein amounts loaded. No double bands were detected in the combined lane suggesting the presence of the same size protein product, cleaved, mature UhAVR1-SP:GFP.

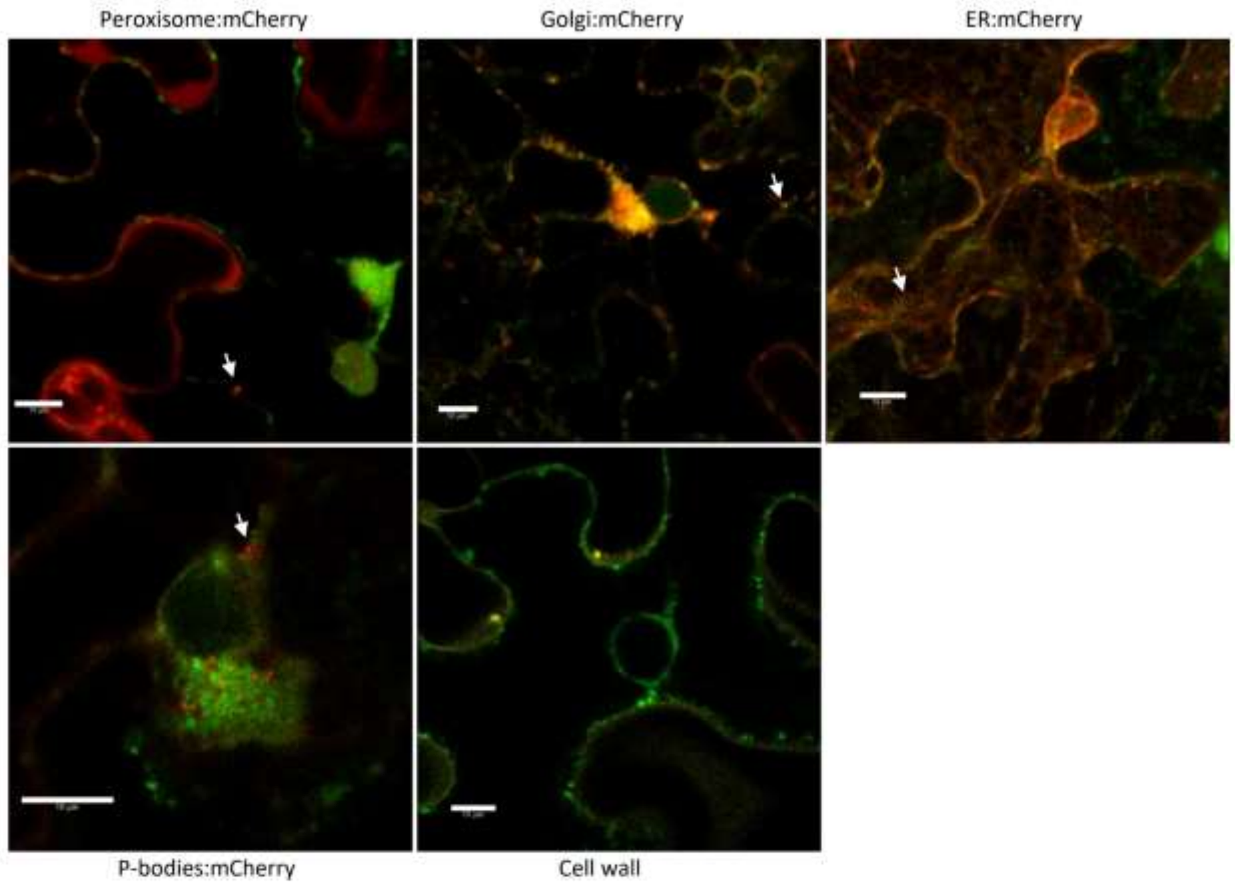

Figure S7. UhAVR1 does not co-localize with plant organelle markers. mCherry-tagged organelle markers (arrows) were co-expressed with UhAvr1+SP:GFP via agroinfiltration in *N. benthamiana* followed by confocal microscopy. UhAvr1+SP:GFP did not co-localize with the following markers at 24 hpi: peroxisome, golgi, endoplasmatic reticulum (ER) or P-bodies. Visualization of the plant cell wall (yellow) was done at 48 hpi by staining tissues with 0.8 mg/ml propidium iodide (PI) for 15 min. UhAvr1+SP:GFP fluorescence is seen inside the cell wall. Two independent experimental repeats were performed with the mentioned markers. All images are snapshots of a single optical section except for the ER image which is a maximum projection of a z-stack. Scale bar represents 10 μm.

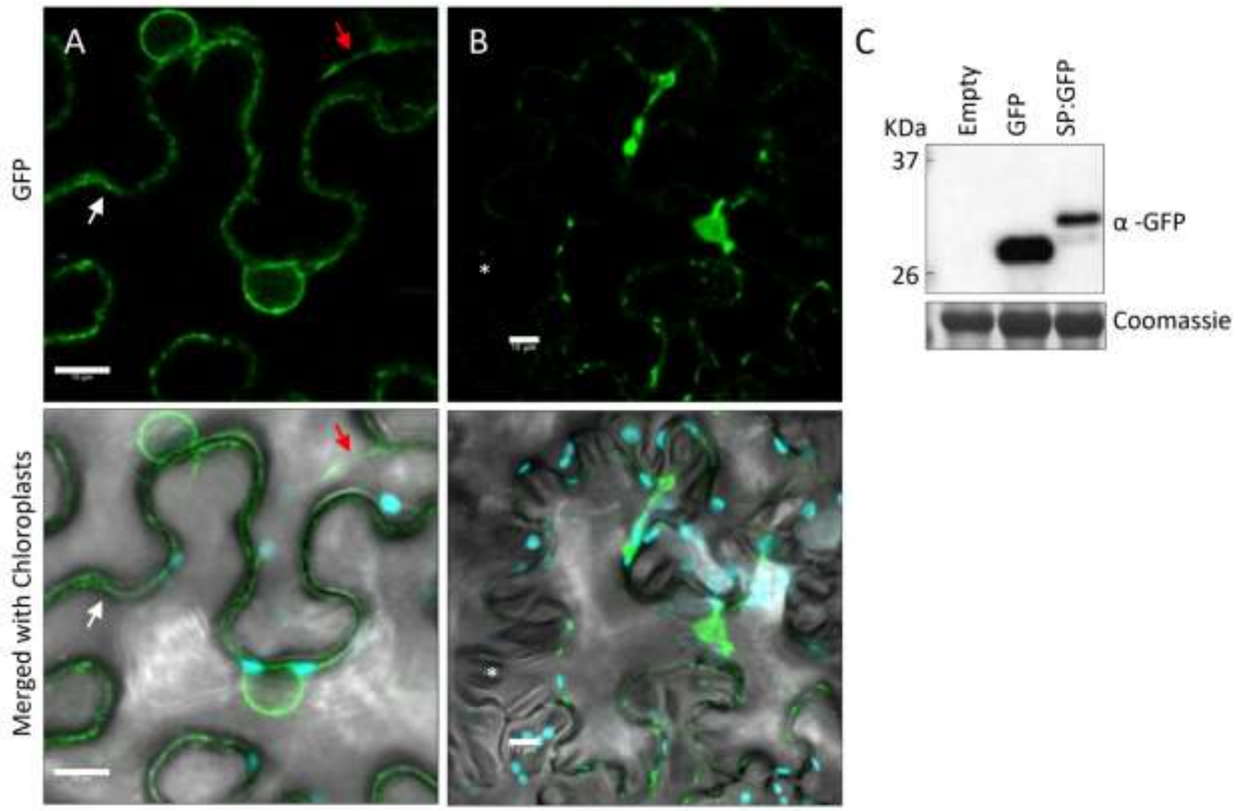

Figure S8. **GFP expressed with the N-terminal SP of UhAVR1 localizes to the cytosol of *N. benthamiana* at 24 hpi.** **(A)** Confocal microscopy of *N. benthamiana* leaves agroinfiltrated with SP:GFP showed GFP fluorescence localizing to the cytosol (red arrow) and cytosolic foci of different sizes (white arrow). **(B)** No fluorescence was seen in the apoplastic space of *N. benthamiana* (asterisk) after plasmolysis was induced with 1.5M NaCl for 20 min. **(C)** Protein blot analysis performed using proteins from the same batch of plants used for panel A showed expressed GFP (27 kDa). Both full length (31 kDa) and cleaved (29 kDa) products were detected from the agro-infiltrated SP:GFP construct. All images are snapshots of a single optical section and the scale bar represents 10 μm.

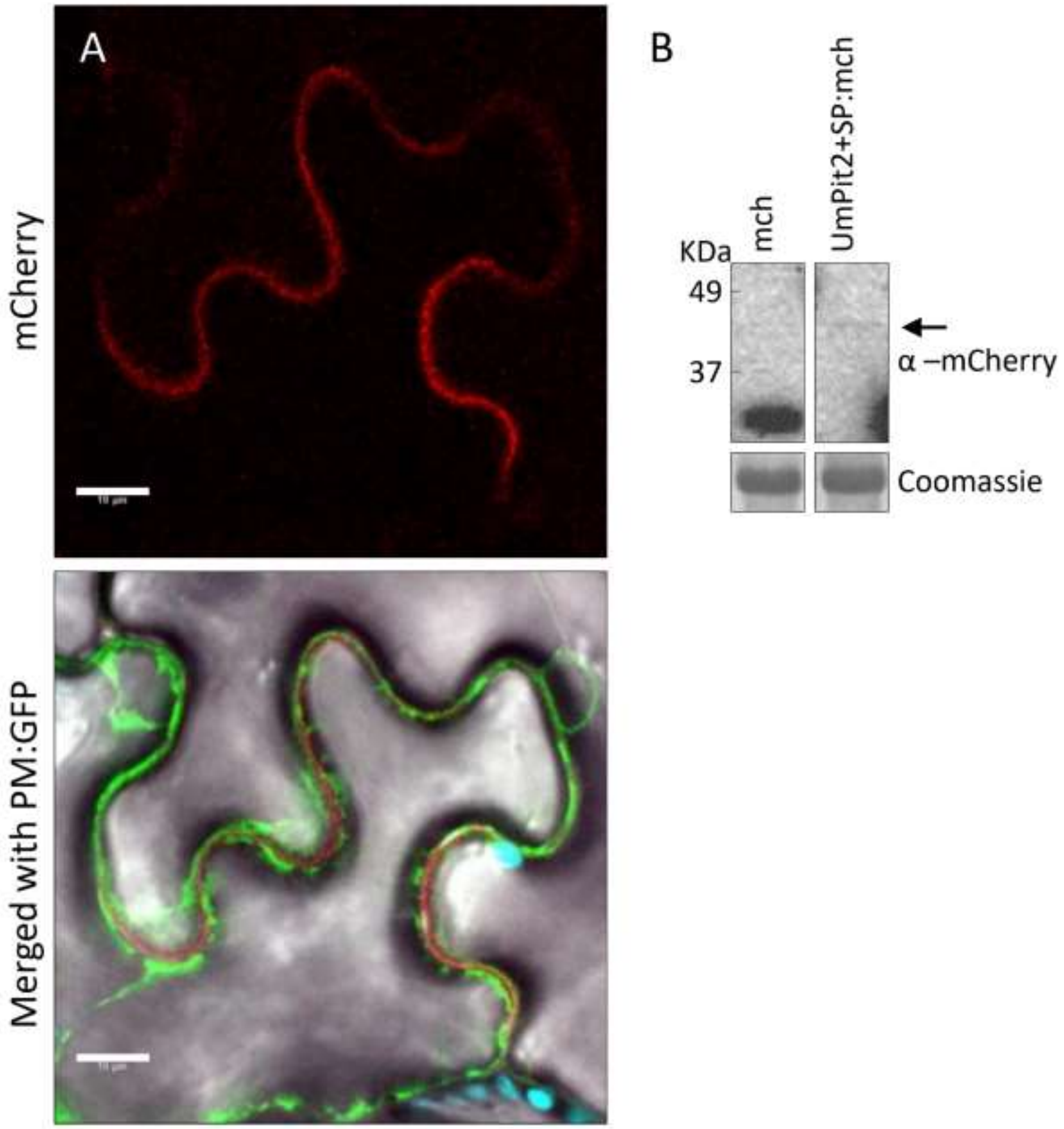

Figure S9. **UmPit2:mCherry localizes to the apoplast in leaves of *N. benthamiana*.** Leaves of *N. benthamiana* plants were co-agroinfiltrated with constructs expressing UmPit2+SP:mCherry and a GFP tagged plasma membrane (PM) marker. **(A)** Confocal microscopy at 24 hpi showed the mCherry fluorescence localizing in between the GFP signals. Images are a snapshot of a single optical section and scale bar represents 10 μm. **(B)** Protein blot analysis of the samples isolated from the same batch of plants used in panel A showed the presence of mCherry (predicted size of 27 kDa) and SP-cleaved UmPit2:mCherry (predicted size of 37 kDa) (arrow). The image originates from the same blot.

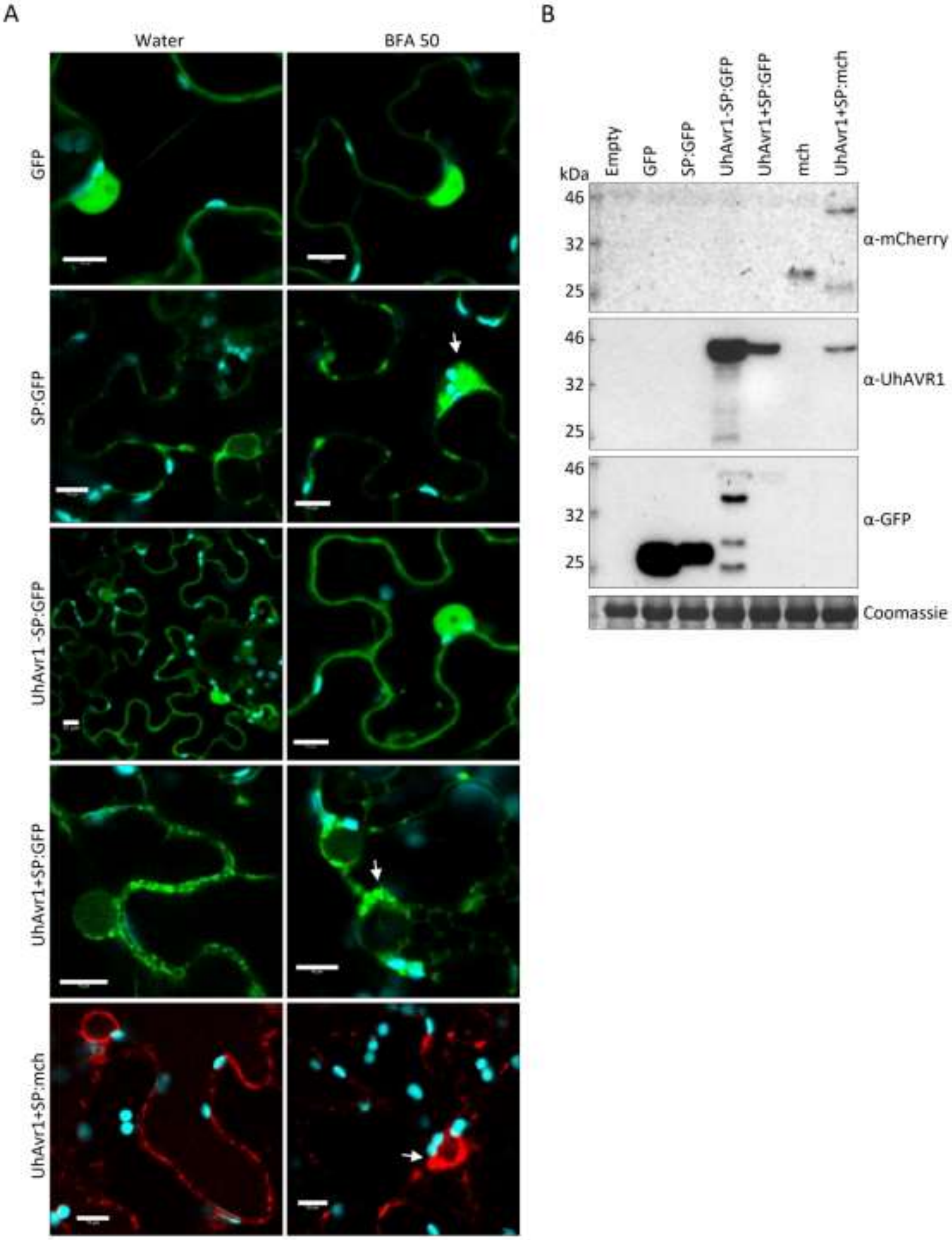

Figure S10. **Brefeldin A exposure in *N. benthamiana* blocks trafficking of proteins with the UhAVR1 SP. (A)** *N. benthamiana* plants transiently expressing fluorescence tagged proteins were exposed to Brefeldin A (BFA) or water. Confocal imaging showed that the constructs with a signal peptide (SP:GFP, UhAvr1+SP:GFP and UhAvr1+SP:mCherry) presented protein aggregation (arrows) only upon exposure to BFA. Constructs lacking a SP (GFP and UhAvr1-SP:GFP) did not show protein aggregation upon water or BFA exposure. All images are snapshots of a single optical section and the scale bar represents 10 μm. **(B)** Protein blot analysis of proteins isolated from the plants used in panel A, prior to Brefeldin A or water exposure, showed the presence of mainly full-length proteins.

Table S1. Fungal strains used in this study

Strain ID	Genotype	Comments
Um001	*a2b2*	Wild type *U. maydis* strain, alias Um518; [1].
Um002	*a1b1*	Wild type *U. maydis* strain, alias Um521; [1].
Uh362	*MAT-2 Uhavr1*	Wild type *U. hordei* strain, alias Uh4854-10; [2].
Uh364	*MAT-1 UhAvr1*	Wild type *U. hordei* strain, alias Uh4857-4; [2].
Uh1289	Uh364 (*MAT-1 ΔUhAvr1*), carboxin^r	*UhAvr1* deletion strain, [3].
Uh1351	Uh364 (*MAT-1 UhAvr1 [otef:gfp]*), zeocin^r	Control strain that constitutively expresses a genome-integrated otef:GFP; [3].
Uh1353	Uh1289 (*MAT-1 ΔUhAvr1* [*UhAvr1:GFP*]), zeocin^r	*UhAvr1* deleted strain expressing *UhAvr1:GFP* from the *UhAvr1* promoter. Chimer is located in *UhAvr1* endogenous location; [3].
Uh1357	Uh1289 (*MAT-1 ΔUhAvr1* [*otef:UhAvr1:GFP*]), zeocin^r	*UhAvr1* deleted strain expressing *UhAvr1:GFP* from the otef promoter. Chimer is located in *UhAvr1* endogenous location; [3].
Uh1397	Uh1289 (*MAT-1 ΔUhAvr1* [*SP:GFP:UhAvr1*]), zeocin^r	*UhAvr1* deleted strain expressing *SP:GFP:UhAvr1* from its *UhAvr1* promoter. Chimer is located in *UhAvr1* endogenous location. Clone 1. This work.
Uh1398	Uh1289 (*MAT-1 ΔUhAvr1* [*SP:GFP:UhAvr1*]), zeocin^r	*UhAvr1* deleted strain expressing *SP:GFP:UhAvr1* from its *UhAvr1* promoter. Chimer is located in *UhAvr1* endogenous location. Clone 2. This work.
Uh1399	*Uh1289 (MAT-1 ΔUhAvr1 [SP:GFP:UhAvr1]), zeocin^r*	*UhAvr1* deleted strain expressing *SP:GFP:UhAvr1* from its *UhAvr1* promoter. Chimer is located in *UhAvr1* endogenous location. Clone 3. This work.
Uh1430	Uh1351 (*MAT-1 UhAvr1 [UhAvr1+SP:mCherry]*), carboxin^r zeocin^r	A strain expressing *UhAvr1+SP:mCherry* in pUHESdest episomal plasmid along with genome-integrated otef:GFP. This work.
Uh1434	Uh1351 (*MAT-1 UhAvr1 [ΔSP:UhAvr1:mCherry]*), carboxin^r zeocin^r	A strain expressing *ΔSP:UhAvr1:mCherry* in pUHESdest episomal plasmid along with genome-integrated otef:GFP. The SP was deleted from pUHESdest vector backbone. This work.
Uh1440	Uh1351 (*MAT-1 UhAvr1 [UmPit2+SP:mCherry]*), carboxin^r zeocin^r	A strain expressing *UmPit2+SP:mCherry* with its SP in pUHESdest episomal plasmid along with genome-integrated otef:GFP. This work.
WT teliospores	Uh362 x Uh364 (*MAT-2 MAT-1 Uhavr1 UhAvr1*)	This work.
UhAvr1m teliospores	Uh362 x Uh1289 (*MAT-2 MAT-1 Uhavr1 ΔUhAvr1*)	This work.
GFP teliospores	Uh362 x Uh1351 (*MAT-2 MAT-1 Uhavr1 UhAvr1 [otef :gfp]*)	Described in [3].
SGUhAvr1 teliospores	Uh362 x Uh1398 (*MAT-2 MAT-1 Uhavr1 ΔUhAvr1* [*SP:GFP:UhAvr1*])	*SP:GFP:UhAvr1* is driven by UhAvr1 wild type promoter. This work.
UhAvr1GFP teliospores	Uh362 x Uh1357 (*MAT-2 MAT-1 Uhavr1 ΔUhAvr1* [*otef:UhAvr1:GFP*])	*SP:GFP:UhAvr1* is driven by otef constitutive promoter; [3].

Table S2. Oligonucleotides used in this work

No.	Primer Name	Sequence (5′-3′)	Purpose
520	Ta_ubi-conj_fw	AGCATTTGCCTTGACATTCTCA	Primer pairs for amplification of barley reference gene *HvUbiq* (*ubiquitin-conjugating enzyme-specific*).

521	Ta_ubi-conj_rev	CCCGATCAGTCTTGTACATG TGA	
1247	UH_10022_fw	CACCATGCGATCGTTTTCCC TTTTCC	Forward primer to generate entry vector of the SP of *UhAvr1*. In red are CACC sequences for directional cloning into the GateWay™ entry vector.
1248	UH_10022-SP_ Fw	CACCATGCCTGGCGACAAA GCTTCTTC	Forward primer to generate entry clone UhAvr1-SP:mCherry. In red are CACC sequences for directional cloning into the GateWay™ entry vector.
1504	mCherry_fw	CACCATGGTGAGCAAGGGC GAGGAGG	Primer pairs to generate entry clone mCherry. In red are CACC sequences for directional cloning into the GateWay™ entry vector.
1505	mCherry_rev	CTTGTACAGCTCGTCCATGC C	
1764	recombined pVSP EcoRV site 1_fw	CCGGATATCACAAGTTTG	Primers to generate *Pseudomonas* vector pVSPΔSPΔRFC-A, where the *Pseudomonas* SP and GateWay™ cassette A have been deleted. Restriction sites are underlined.
1765	recombined pVSP EcoRV site1_rev	CCGGATATCACCACTTTGTA C	
1770	pVSP frag1 5′ BglII_fw	GAAGATCTGTAGATTTGTGA TGG	
1771	pVSP frag2 3′ BglII_rev	GAAGATCTTGATCCCCTG	
1776	pVSP-EcoRV frag 2 5′EcoRV_fw	GATCGATATCGGATCGATC CTATCCGTACG	
1777	pVSP-EcoRV frag 1 3′EcoRV_rev	CGCGATATCCATAAAAAAC C	
1794	SalI_3′ flank Uh10022_fw	GGCGTCGACCTTAGCCTAGT CCCGCTCT	3′ flanking primer to generate *UhAvr1* gene probe. SalI site is underlined
1795	SpeI_3′ flank Uh10022_rev	GGCACTAGTGAGAAGAAGC AGGGCTTTCA	3′ flanking primer to generate *UhAvr1* gene probe. SpeI site is underlined
1965	UhGapdh-fw	CAAGGCTCAGATCGTCTCC A	Primer pairs to amplify *U. hordei* fungal reference gene *UhGapdh* (*glyceraldehyde 3-phosphate dehydrogenase*)
1966	UhGapdh-rev	GGATGATGTTGGCAGCAGC G	
1967	Uh10022_ ddPCR1_fw	AGTAGACAGGAATCTTGCC TGG	Primer pairs to quantify *UhAvr1* expression in ddPCR
1968	Uh10022_ ddPCR1_rev	GGTCAGAACGTCTCCAATCT CG	
2031	BamHI_Avr1+SP_ fw	GGAGGCGGATCCAACCATG CGATCGTTTTCCCTT	Primer pairs to clone *UhAvr1+SP* into pCAMBIA:mCherry vector. BamHI site is underlined
2032	Avr1+SP_BamHI_ rev	TAAAACGGATCCACCTCCT CCGGCAAATCGGAGCGC	
2034	mCherry_STOP_ rev	**TTA**CTTGTACAGCTCGTCCA TGCC	Reverse primer to generate entry clone UhAvr1-SP:mCherry. The natural stop codon is in bold.
2035	SPUhAvr1_rev	AGA AGC TTT GTC GCC AGG TGC	Reverse primer to generate entry vector of the SP of *UhAvr1*.
2098	pUHes_UhAvr1m CherryΔSP_fw	GGCGAATTCTGATATCACA AGTTTGTACAAAGCAGG	Outward-facing primers to delete the SP of the pUHESdest vector backbone by inverse PCR. EcoRI site is underlined.
2099	pUHes_UhAvr1m CherryΔSP_rev	CCGGAATTCATTGCCCCCCG GGATCTG	
2125	pUHES_gib_fw	TGACGCGCGGTTTGACTTGT C	Primer pairs to amplify pUHESdest for cloning *UmPit2+SP:mCherry* via Gibson assembly.
2126	pUHES_gib_rev	TGCCCCCCGGGATCTGGC	

2127	UmPit2+SPmCherry_gib_fw	TTGCCAGATCCCGGGGGGCA**ATG**CTGTTTCGCTCAGCC	Primer pairs to amplify and clone *UmPit2+SP:mCherry* into pUHESdest via Gibson assembly. Start and stop codons are in bold.
2128	UmPit2+SP mCherry_gib_rev	ACAAGTCAAACCGCGCGTCA**TTA**CTTGTACAGCTCGTCCATG	
2129	BamHI_UmPit2+SP_fw	GGAGGCGGATCCAACC**ATG**CTGTTTCGCTCAGCC	Primer pairs to clone *UmPit2+SP* into pCAMBIA: mCherry vector. BamHI site is underlined and the start codon is in bold.
2130	UmPit2+SP_BamHI_rev	TAAAACGGATCCACCTCCTTCCCAGATGACCACATCTCCGT	
2174	GFP5'-ClaI-fw	AGGTCAATCGAT**ATG**GTGAGCAAGGGCGAGG	Primer pairs to clone GFP into FoMV PV101. ClaI and XbaI site is underlined. Start and stop codon are in bold.
2175	GFP3'-XbaI-rev	TATGCTTCTAGA**TTA**CTTGTACAGCTCGTCCATG	
2176	UhAVR1_Cla1-fw	AGGTCAATCGAT**ATG**CGATCGTTTTCCCTTTTCCTC	Forward primer to clone *UhAvr1+SP* into FoMV PV101. ClaI site is underlined and the start codon is in bold.
2177	UhAVR1-SP_Cla1-fw	AGGTCAATCGAT**ATG**CCTGGCGACAAAGCTTC	Forward primer to clone *UhAvr1-SP* into FoMV PV101. ClaI site is underlined and the start codon is in bold.
2178	UhAVR1_XbaI-rev	TATGCTTCTAGA**TCA**TCCGGCAAATCGGAGCG	Reverse primer to clone *UhAvr1* into FoMV PV101. XbaI site is underlined and the natural stop codon is in bold.
2224	Nb_PP2A_fw	GACCCTGATGTTGATGTTCGCT	Primer pairs to amplify *Nicotiana benthamiana* reference gene *PP2A* (*protein phosphatase 2*).
2225	Nb_PP2A_rev	GAGGGATTTGAAGAGAGATTTC	

Table S3. *UmPit2* effector mRNA sequence with SP (underlined) and STOP codon

AtgctgtttcgctcagcctttgttctgctcatcgtggcctttgcaagtgcatgcctggtgcaacatgttcaagctattccggtgcgtcgatcgctctctaccgatgcctcaatgagctcggctgctggcaagctcaaccggagatggtggttcggcttcacaggttcgctcggcaaggaacctgacaacggccaagtacagatcaagatcatcccagacgcgctcatcatcaagaatccgcctgccaacaaagacgatctgaacaagctaatcgaaaacctaaaacgcaagcacccaagattcaagacggtggtcatgccgacagatcctaacggagatgtggtcatctgggaaTAA

Table S4. Peptide sequence used to generate the UhAVR1 antibody

Anti-UhAVR1 antibody was produced from only 155 amino acids sequences of UhAVR1:
PSFKLEIAENPNVDPFLEKISKLGNSHDLYPHVALMRTTLYGKDKLTTNLGAYPDFRRFIYLGNSPGVPEMYFAVPLHLNPHGVDRNLAWSLIYAHSDQPKTLVHHGFVSASGGHLVLDKVKKTNYPSSRSFEIGDVLTLREILDIELPALRFAG.

Table S5. Pathogenicity assays of N-terminally GFP tagged *UhAvr1* strains showed loss of avirulence

***U. hordei* (*Uh*) strains crossed**	**Barley cv.**	**No. of plants diseased (%)**	**No. of plants inoculated**
Uh362 (*MAT-2, Uhavr1*) x Uh364 (*MAT-1, UhAvr1*)	Odessa	32 (51)	63
	Hannchen	0 (0)	61
Uh362 (*MAT-2, Uhavr1*) x Uh1289 (Uh364 (*MAT-1 ΔUhAvr1*))	Odessa	21 (46)	46
	Hannchen	21 (43)	49
Uh362 (*MAT-2, Uhavr1*) x Uh1397 (Uh1289 (*MAT-1 ΔUhAvr1* [*SP:GFP:UhAvr1*])	Odessa	23 (43)	53
	Hannchen	26 (46)	57
Uh362 (*MAT-2, Uhavr1*) x Uh1398 (Uh1289 (*MAT-1 ΔUhAvr1* [*SP:GFP:UhAvr1*])	Odessa	22 (34)	65
	Hannchen	13 (19)	69
Uh362 (*MAT-2, Uhavr1*) x Uh1399 (Uh1289 (*MAT-1 ΔUhAvr1* [*SP:GFP:UhAvr1*])	Odessa	30 (54)	56
	Hannchen	20 (42)	48

Supplementary references

1. Kronstad, J.W.; Leong, S.A. Isolation of two alleles of the *b* locus of *Ustilago maydis*. *Proc Natl Acad Sci USA* **1989**, *86*, 978-982.
2. Linning, R.; Lin, D.; Lee, N.; Abdennadher, M.; Gaudet, D.; Thomas, P.; Mills, D.; Kronstad, J.W.; Bakkeren, G. Marker-based cloning of the region containing the *UhAvr1* avirulence gene from the basidiomycete barley pathogen *Ustilago hordei*. *Genetics* **2004**, *166*, 99-111.
3. Ali, S.; Laurie, J.D.; Linning, R.; Cervantes-Chávez, J.A.; Gaudet, D.; Bakkeren, G. An Immunity-Triggering Effector from the Barley Smut Fungus *Ustilago hordei* Resides in an Ustilaginaceae-Specific Cluster Bearing Signs of Transposable Element-Assisted Evolution. *PLOS Pathogens* **2014**, *10*, e1004223.
